# A four-year-old Nigerian boy with battered child syndrome: implications for public health

**DOI:** 10.11604/pamj.2020.35.47.19115

**Published:** 2020-02-18

**Authors:** Olayinka Rasheed Ibrahim, Fatima Faskari Nasir, Abubakar Sani Lugga, Mu'utasim Ibrahim

**Affiliations:** 1 Department of Pediatrics, Federal Medical Centre, Katsina, Katsina State, Nigeria

**Keywords:** Battered child syndrome, child abuse, Nigerian, public health

## Abstract

Battered child syndrome (BCS) is a form of physical abuse that is characterised by multiple injuries and potentially fatal outcome. Despite the high prevalence of physical abuse in developing countries, BCS is rarely reported. Hence, this report highlighted a four-year-old Nigerian boy who suffered multiple injuries (scalp haematoma, bruises, right clavicular fracture, and burns) from the paternal uncle’s wife. This case report is discussed along the line of public health approach for curbing the social menace.

## Introduction

Battered child syndrome ((BCS) is a form of physical abuse characterised by multiple injuries and potentially fatal outcome. It is defined as the collection of injuries sustained by a child as a result of repeated mistreatment or beating [1]. Although description classical of this form of maltreatment existed in the literature, it was Kempe and his colleagues at the Colorado University that introduced the term in a landmark article in 1962 [2]. The paper not only drew attention to this form of physical abuse but also other forms of abuse, which led into actions at various countries and at the global arena [3]. Whereas Child abuse is a global problem, it has been an under-researched area and under-reported in the developing countries where the prevalence is believed to be high [4]. Indeed, the first series of researches and case reports were documented from the developed countries [3]. Thus, it was not surprising that Nigeria only recently carried out a national survey on the violence against children which found that about 50% of the respondents have been subjected to some form of physical abuse in their childhood [5]. The survey also observed that among those that have been subjected to physical abuse, 50% have reported to someone, but only 5% received help. This survey further buttresses the under-reporting of cases of child abuse including battering in Nigeria [5]. The survey findings are not unexpected, considering the various sociocultural issues that permit and abate child abuse in Nigeria, under the pretense of child discipline [6]. The Nigerian government was a prompt signatory to the African Charter on the Rights and Welfare of the Child 1989 and United Nations Convention on the Rights of the Child 1990, although the country was only able to sign into law the Nigerian Child’s Rights Act (CRA) in 2003 [7]. The CRA has been domesticated in most states of the Nation [8], however, the action at individuals’ level, family and community levels are still far behind in curbing this menace. Hence, this paper highlighted a case of a 4-year-old boy who suffered abusive head trauma (scalp haematoma), bruises, rope signs on the skin, right clavicular fracture and burns from the caregiver (paternal uncle’s wife). This is discussed along the line of public health approach, which is in keeping with the global calls toward a halt of child abuse and ensure all children are protected from violence [9, 10].

## Patient and observation

A 4-year-old boy was referred to the Emergency Paediatric Unit of the hospital with the complaint of forehead swelling of a day duration. He sustained the injury while being beaten by the paternal Uncle’s wife (which she initially denied). Further enquiries revealed that the child had been subjected to physical abuse in the preceding few weeks, during which he had a right clavicular fracture. He also sustained hot water scald burns injury close to the location of the clavicular fracture. The boy had lost his father about three months before presentation and was relocated to the paternal uncle and his wife as the new caregivers.

**Figure 1:**
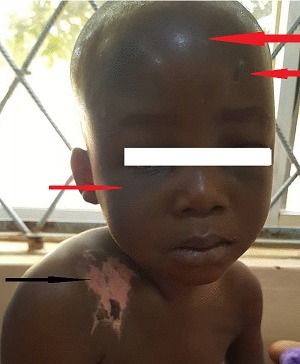
A 4-year-old with forehead swelling, bruises, darkish discolouration around the lower part of the eyes (Panda eyes sign) (red arrows) and burns on the right shoulder (black arrow)

**Figure 2:**
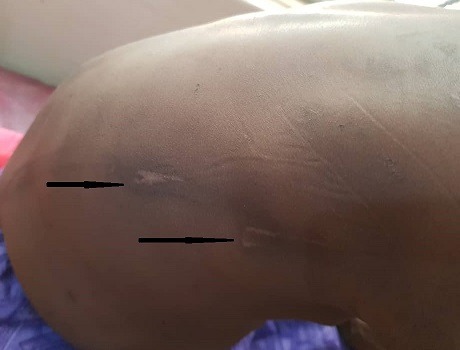
“Rope” sign and scars on the back (black arrows)

**Figure 3:**
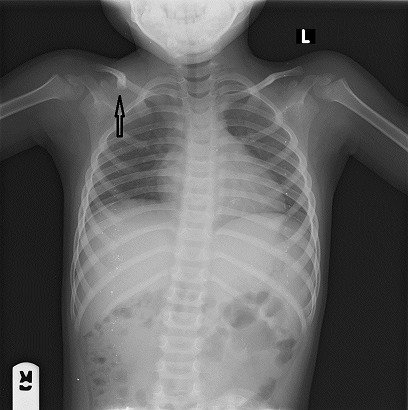
Chest radiography showing right clavicular fracture (black arrow)

**Figure 4:**
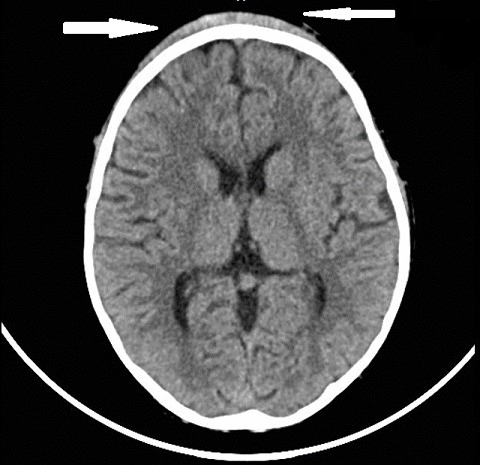
A computerized tomography showing frontal scalp haematoma (white arrows)

At presentation, he was conscious with forehead swelling measuring about 8 cm x 8 cm, soft, fluctuant, tender (Figure 1) and darkish discolouration around the lower part of the eyes (Panda eyes sign). He also had swelling over the right clavicle, hard and tender with an area of healing scald burns injury (about 6 cm x 5 cm) lateral to it (Figure 1), multiple bruises at various stages of healing and ‘rope marks’ over the back and extremities (Figure 2). The other systemic examinations findings were not remarkable. The X-ray of the shoulder showed right clavicular fracture with callus formation (Figure 3). The computerised tomography scan of the cranium showed frontal scalp haematoma and normal intracranial findings (Figure 4).

A diagnosis of battered child syndrome was made; he was admitted and placed on analgesia and antibiotics. The orthopaedic surgeons reviewed and opted to manage the fractures conservatively (non-operative). Social welfare department traced the home, got the community leaders involved, and a report was also made to the law enforcement agents. The child was discharged home on the fifth day and allowed to be reunited with his biological mother. Before being reunited with the mother (a week after discharge), a regular home visit was carried out by the social welfare who confirmed the cessation of further abuse. Furthermore, the boy was also seen on a scheduled clinical appointment with no evidence of further abuse.

## Discussion

In Nigeria, perhaps this is the first case report of BCS in a child aged less than five years as a search of the major database (PubMed, Embase, Scopus, Web of Science, Google scholar and AJOL) revealed none. Indeed, a review of local literature in Nigeria revealed that most of the few reported cases of child abuse were in adolescents with predominance of sexual abuse cases [11]. This case also illustrates one of the several child abuse ongoing in the society, most of whom are probably unreported and undocumented with potential attendant short-term and long-term complications [12, 13].

There is no doubt about the good intention of the Nigerian government against child abuse with signatory to the various convention on the rights of the child, but the country needs to take a pragmatic approach towards the implementation of the various laws that protect them. Furthermore, there is a need to ensure that all states in Nigeria domesticate CRA, a major stumbling blog to the implementation of the law [14]. The government needs to ensure that the various actions are coordinated; her citizens are aware of the law and what to do where a case of child abuse is being suspected. Besides, bringing the culprits to book through a successful prosecution based on the existing law will also serve as a deterrent to the perpetrators [7]. The actions will be in keeping with the first point (implementation and enforcement of laws) of ‘INSPIRE’; a seven-point strategies by the World Health Organization (WHO) towards ending violence against children and achievement of Sustainable Development Goals (SDG) target 16.2, end abuse, exploitation, trafficking and all forms of violence against and torture of children by 2030 [15].

The United Nations Children’s Fund (UNICEF) is spearheading the collaborations between the various international organisations, and the Government of Nigeria and one of the aftermaths is the recent launch of a recent survey on child abuse in Nigeria [5]. Besides the recent survey, there is still a need to conduct a large nation-wide in-depth study that will identify the burden of the problem, especially in the younger children less than five years, whom studies have shown bear a greater impact of child abuse [16]. The younger age groups were not captured in the survey [5]. Although few local studies documented the sociocultural issues as possible risk factors, there is a need to identify other critical factors including access to help, that may be a hindrance toward Nigeria achieving SDG 16.2. Thus, public health approach in Nigeria will then stem from identification of actual burden of child abuse, identified various associated factors, design and implementation of strategies that will address the menace. There is also a need for a structured program that will re-appraise various interventions from time to time, in order to strengthen effective measures.

The family unit remains one the critical components in the child abuse. Indeed, some of the programs such as ‘family-nursing partnership’ in the developed countries have been found successful [17]. A similar program in Liberia was also successful in reducing child abuse [17]. It is high time Nigeria also adopts and implements such a program. The program may be well accepted if it incorporates the community leaders and religious leaders. The program can also be used to enlighten members of the public on child abuse, further raise awareness on what constitutes child abuse especially in the socio-cultural issues and provide correct information to the members of the public.

## Conclusion

This case report highlighted the threats of abuse a child face as he or she grows in a developing country like Nigeria, where unfortunately most go unnoticed, and unreported. This case report also suggests the need to carry out a nation-wide in-depth study to determine the burden of child abuse, identify the country peculiarity and proffer solutions based on the identified issues.

## Competing interests

The authors declare no competing interests.
